# A Highly Selective Turn-on Fluorescent Probe for the Detection of Aluminum and Its Application to Bio-Imaging

**DOI:** 10.3390/s19112423

**Published:** 2019-05-28

**Authors:** Liguo Wang, Jing Yang, Huan Wang, Chongzhao Ran, Ying Su, Long Zhao

**Affiliations:** 1 Institute of Evolution and Marine Biodiversity, Ocean University of China, Qingdao 266003, China; wlg159543@163.com (L.W.); suying@ouc.edu.cn (Y.S.); 2 Athinoula A. Martinos Center for Biomedical Imaging, Massachusetts General Hospital and Harvard Medical School, Boston, MA 02129, USA; hwang59@mgh.harvard.edu (H.W.); cran@nmr.mgh.harvard.edu (C.R.); 3 Cardiovascular Research Center, Massachusetts General Hospital and Harvard Medical School, Boston, MA 02129, USA

**Keywords:** aluminum, fluorescent probe, bio-imaging

## Abstract

Aluminum is the most abundant metallic element in the Earth’s crust and acts as a non-essential element for biological species. The accumulation of excessive amounts of aluminum can be harmful to biological species. Thus, the development of convenient and selective tools for the aluminum detection is necessary. In this work, a highly selective aluminum ion fluorescent probe N’-(2,5-dihydroxybenzylidene)acetohydrazide (Al-II) has been successfully synthesized and systemically characterized. The fluorescence intensity of this probe shows a significant enhancement in the presence of Al^3+^, which is subject to the strong quench effects caused by Cu^2+^ and Fe^3+^. The binding ratio of probe-Al^3+^ was determined from the Job’s plot to be 1:1. Moreover, the probe was demonstrated to be effective for in vivo imaging of the intracellular aluminum ion in both living *Drosophila* S2 cells and Malpighian tubules.

## 1. Introduction

Aluminum is the most abundant metallic element in the Earth’s crust. It can be found in a wide range of plants, drinking water, and food. It is also commonly used as metallic materials in the industrial and medical fields [1,2,3]. However, aluminum is a non-essential element for the human body. The accumulation of excessive amounts of aluminum can be harmful to human bodies, causing many medical problems and illnesses, including neurodegenerative and neurological disorders, such as Alzheimer’s disease or Parkinson’s disease [3,4,5,6,7].

Compared with other transition metal ions, the detection of aluminum has always been challenged by its poor coordination ability, strong hydration tendency, and the lack of spectroscopic characteristics [8]. Therefore, it is urgent to develop the convenient and selective methods for the detection of aluminum. Fluorescent approach is the most attractive and highly sensitive method to detect low concentrations of analysts [9]. In recent years, there has been considerable progress for the detection of aluminum utilizing fluorescent chemo-sensors [10,11,12,13,14,15]. However, there are still many limitations during the application of most reported fluorescent probes, such as working well only in anhydrous systems, poor selectivity or sensitivity, and weak binding ability [13,16,17,18,19]. Especially, the chemo-sensors for biological system are rare, most of which were applied in in vitro cultured cells, but not the living organisms. We summarized the reported Al probes suitable for bio-imaging in Table 1.

In this work, we synthesized a highly selective turn-on fluorescent probe N’-(2,5-dihydroxybenzylidene)acetohydrazide (Al-II) for Al^3+^ detection, which worked well in aqueous systems. Moreover, we successfully applied this probe to monitor the in vivo distribution of Al^3+^ either in living cultured *Drosophila* cells or organs, which provides a useful tool to selectively detect aluminum not only in inorganic systems but also in biological systems.

## 2. Materials and Methods

### 2.1. HPLC Methods

Liquid chromatography-mass spectrometry (LC-MS) was performed using an Agilent 1200 Series apparatus with an LC/MSD trap and Daly conversion dynode detector with UV detection at 254 nm. The reverse phase high pressure liquid chromotragphy (RP-HPLC) method used in compound characterization is as follows: (A) Luna C18 column (100 × 2 mm); eluent A: H_2_O/0.1% formic acid, B: MeCN/0.1% formic acid; gradient: 5% B to 95% B over 3 min, 95% B for 1.5 min, 95% B to 5% B for 0.5 min, then 5% B for 2 min; flow rate 0.7 mL/min. (B) Luna C18 column (100 × 2 mm); eluent C: 10 mM ammonium acetate, D: 90% MeCN/10% 10 mM ammonium acetate; gradient 5% D for 0-1 min, 5 to 95% D from 1-11 min, 95% D from 11-12 min, 95% to 5% D from 12-13 min, 5% D from 13-15 min; flow rate 0.7 mL/min.

### 2.2. Synthesis of the Probe

N’-(2,5-dihydroxybenzylidene)acetohydrazide was prepared by the refluxing acetic hydrazide (0.37 g, 5.0 mmol) with salicylaldehyde (0.69 g, 5.0 mmol) in 50 mL methanol for 3 h. The solution was concentrated under reduced pressure, and the residue was further purified by recrystallization from methanol to afford 0.87 g (90%) as light green crystals.

LC-MS (method B): t_R_ = 4.462 min, m/z = 195.0 [M+ H]^+^ (Figure S1).

^1^H NMR (major:minor isomer = 1:0.88) (500 MHz, (CD_3_)_2_SO) δ 11.49 (s, 1H, major), 11.16 (s, 1H, minor), 10.29 (s, 1H, major), 9.39 (s, 1H, minor), 8.94 (s, 1H, major), 8.88 (s, 1H, minor), 8.24 (s, 1H, major), 8.19 (s, 1H, minor), 7.03 (d, J = 3.0 Hz, 1H, minor), 6.91 (d, J = 2.6 Hz, 1H, major), 6.74–6.61 (m, 4H, 2 from major, 2 from minor), 2.16 (s, 3H, minor), 1.95 (s, 3H, major) (Figure S2).

^13^C NMR (126 MHz, (CD_3_)_2_SO) δ 171.45, 165.28, 150.00, 149.92, 149.80, 149.26, 145.55, 140.61, 120.29, 118.92, 118.61, 118.39, 116.93, 113.81, 111.39, 21.40, 20.30 (Figure S3).

### 2.3. Compound Stock and Storage

The Al-II probe powder was kept at 4 °C for long-term storage. The probe was dissolved into dimethyl sulfoxide (DMSO) to make a 10 mM stock solution, which was stored at 4 °C for temporary preservation.

### 2.4. Drosophila S2 Cell Culture and Experiments

*Drosophila* embryonic S2 cells were cultured with Gibco Schneider’s *Drosophila* Medium (Invitrogen) containing 10% fetal bovine serum and penicillin (50 IU/mL)/streptomycin (50 μg/mL) antibiotics at 25 °C.

For the aluminum detection experiments, the S2 cells were treated with Al^3+^-containing culture medium for 30 min, and then washed with PBS buffer three times, followed by the incubation with Al-II probe–containing medium for 30 min and additional PBS wash three times. Finally, the fluorescent signals indicating Al^3+^ ions in these cells were observed under the confocal fluorescence microscope. All steps were carried out at room temperature.

### 2.5. Al^3+^ Detection in Malpighian Tubules

We chose the *Drosophila* larval Malpighian tubules as the in vivo organ system to perform the Al^3+^ detection experiments using the Al-II probe.

*Drosophila* larvae at third instar stage were fed with fly food containing Al^3+^ for 30 min. Then the Malpighian tubules were dissected out from larval bodies, washed with PBS buffer, and further treated with Al-II probe for another 30 min followed by another round of PBS wash. Eventually, the Malpighian tubules were observed under the confocal fluorescence microscope. All steps were carried out at room temperature.

### 2.6. Confocal Fluorescence Imaging

Fluorescence imaging was acquired using a Nikon Ti laser scanning confocal fluorescence microscope with a laser (λ_ex_ = 402 nm). The emission wavelength range was 500–600 nm. The images were captured under 60x oil lens (cells) or 20x lens (Malpighian tubules) with the pinhole size 71.5 μm and Si PMT HV 147.

The fluorescence intensity in cells was measured using the software ImageJ with the threshold (Triangle mode) adjustment. The fluorescence intensity in *Drosophila* Malpighian tubules was measured using the software ImageJ, then was normalized by the value of control group. The statistical significance was evaluated with Student’s t-test.

## 3. Results and Discussion

**Scheme 1:**
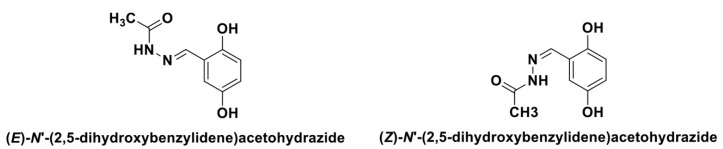
Molecular structure of the Al-II probe.

The desired fluorescent probe Al-II shown in Scheme 1 was synthesized as described above and characterized by LC-MS (Figure S1), ^1^H NMR (Figure S2) and ^13^C NMR (Figure S3).

### 3.1. Fluorescence Spectra of Detecting Al^3+^

**Figure 1:**
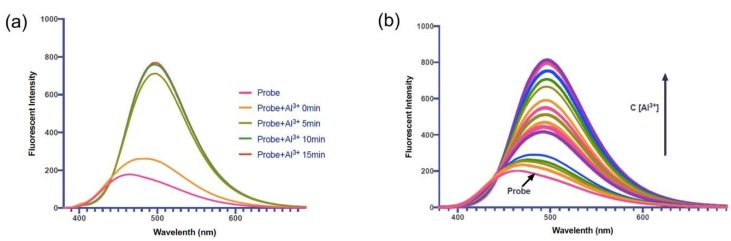
Fluorescent spectra of Al-II probe with Al^3+^. (**a**) Fluorescent spectra of Al-II probe (10 μM) with Al^3+^ (50 μM) after incubation for 0 min, 5 min, 10 min and 15 min. (**b**) Fluorescent spectra of Al-II probe (10 μM) with titration of increased concentration of Al^3+^ (1 μM to 50 μM).

The reaction time on the binding process of the probe with Al^3+^ was firstly studied. As shown in Figure 1a, there was obvious red-shift emission and dramatic intensity increase after addition of Al^3+^ in 5 min and the intensity reached to a stable value in 10 min. So, in the following studies, we pre-incubated probe-metal ions for 15 min before testing. The kinetic constant was estimated to be 0.0036 μM^-1^ min^-1^ based on the linear portion of increase on fluorescent intensity at 500 nm.

Then we performed a detailed investigation on the Al-II probe recognition of Al^3+^. As shown in Figure 1b, the fluorescence intensity of Al-II probe (10 μM) in aqueous solution at 465 nm was dramatically increased with the obvious emission red shift to 500 nm upon addition of Al^3+^. The changes of the emission intensities became constant when the amount of Al^3+^ added reached to 5.0 equiv. (50 μM).

As the emission of Al-II probe itself has spectra contribution to the spectra of probe-Al^3+^, so the spectra of Al-II probe were taken as the background signal and was deducted from probe-Al^3+^ spectra. The relative fluorescent intensity was used for future analysis. The same trend was seen after background deduction (Figure S4). After one-site specific binding analysis, the binding constant of Al-II probe with Al^3+^ was 10.8 μM (Figure S5).

### 3.2. Selectivity Over Metal Ions

**Figure 2:**
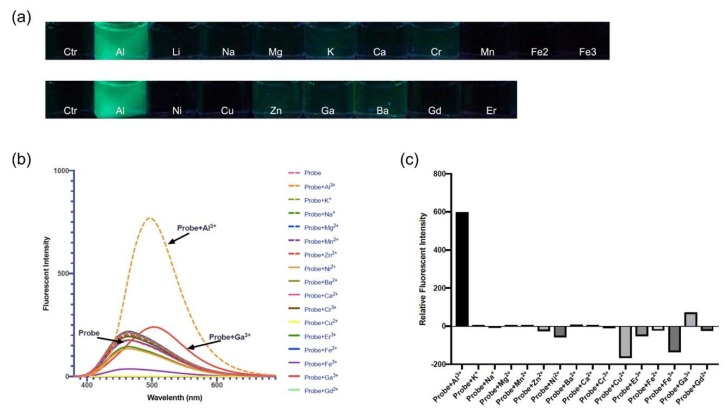
Fluorescence response of the probe Al-II to different metal ions. (**a**) Changes in the fluorescence of probe Al-II (10 μM) upon addition of various metal ions (10 equiv.) under excitation at 365 nm. (**b**) Fluorescent spectra of Al-II probe (10 μM) in the presence of Al^3+^ (50 μM) and various metal ions (50 μM). (**c**) Relative fluorescent intensity of Al-II probe in the presence of Al^3+^ and various metal ions (λ_ex_ 350 nm, λ_em_ 500 nm).

The selectivity of Al-II probe as a fluorescent chemo-sensor for the detection of Al^3+^ among a wide range of environmentally and physiologically active metal ions was investigated by examining the fluorescence of solutions containing Al-II probe and the metal ions in distilled water. As shown in Figure 2a, when 10 equiv. of various metal ions (Al^3+^, Li^+^, Na^+^, Mg^2+^, K^+^, Ca^2+^, Cr^3+^, Mn^2+^, Fe^2+^, Fe^3+^, Ni^2+^, Cu^2+^, Zn^2+^, Ga^3+^, Ba^2+^, Gd^2+^, and Er^3+^) were added to the Al-II probe solution (10 μM), Al^3+^ produced a strong green fluorescence under excitation at 365 nm, whereas other metal ions made no obvious fluorescence.

To more accurately explore the selectivity of probe Al-II over various metal ions, the fluorescence spectra of probe was measured (Figure 2b,c). In the presence of Al^3+^, the fluorescence intensity of Al-II probe showed a large enhancement along with a red-shifted emission. In the cases of other metal ions, such as K^+^, Na^+^, Mg^2+^, Mn^2+^, Zn^2+^, Ba^2+^, Ca^2+^, Cr^3+^, Fe^2+^ and Gd^2+^, there were no apparent changes in the fluorescence spectrum of Al-II probe (Figure 2b). Among these various metal ions, Ga^3+^ also showed an emission red shift, but its intensity was much lower in comparison with Al^3+^ (Figure 2b). Several metal ions such as Ni^2+^, Er^3+^, Cu^2+^ and Fe^3+^, quenched the emission intensity (Figure 2c), which might be attributed to their intrinsic quenching nature. These results indicated a highly selective ability for probe Al-II to detect Al^3+^ ions.

**Figure 3:**
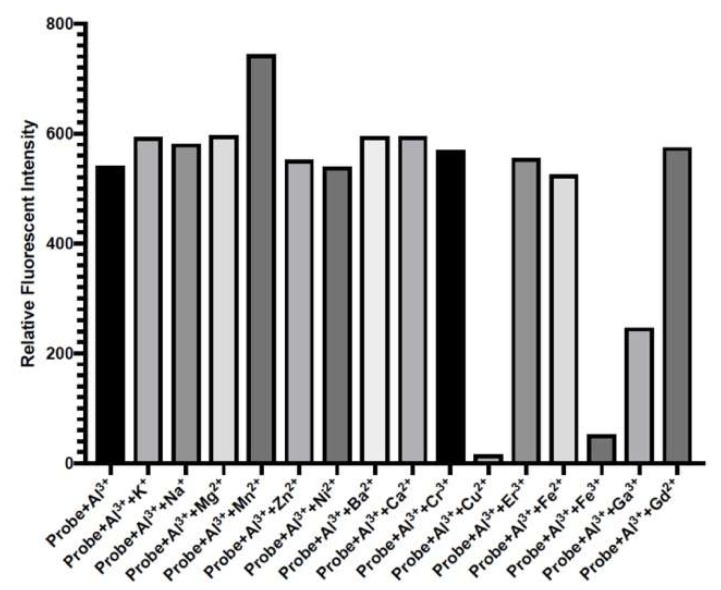
Relative fluorescent intensity of Al-II probe (10 μM) upon addition of Al^3+^ (50 μM) in the presence of various metal ions (50 μM) in aqueous solution (λ_ex_ 350 nm, λ_em_ 500 nm).

To further investigate the binding affinity of Al-II probe with Al^3+^ in the presence of various competing metal ions, Al-II probe was incubated with 5 equiv. of Al^3+^ in the presence of 5 equiv. of other metal ions. There was little interference for the detection of Al^3+^ in the presence of K^+^, Na^+^, Mg^2+^, Mn^2+^, Zn^2+^, Ni^2+^, Ba^2+^, Ca^2+^, Cr^3+^, Er^3+^, Fe^2+^ and Gd^2+^ (Figure 3, Figure S6). The Ga^3+^ ion did have weak competition effect for the Al-II-Al^3+^ binding, while Cu^2+^ and Fe^3+^ ions have strong quench effects for the fluorescence of probe Al-II with Al^3+^.

### 3.3. Detection Range

**Figure 4:**
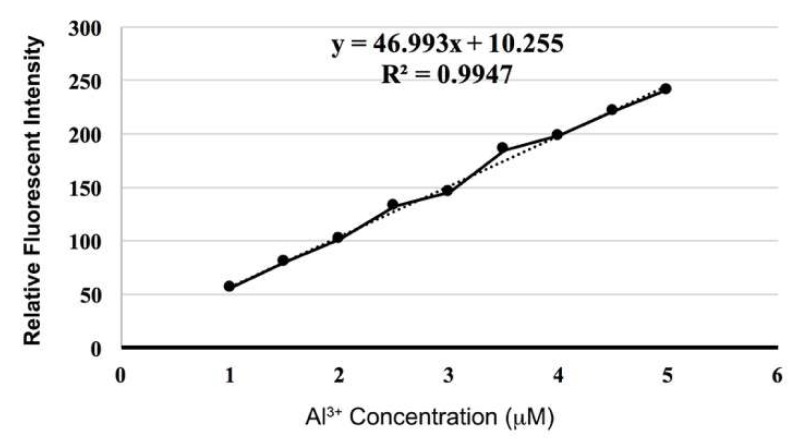
Relative fluorescent intensity of Al-II probe (10 μM) upon addition of Al^3+^ (1 to 5 μM) (λ_ex_ 350 nm, λ_em_ 500 nm).

The detection limit was calculated based on the fluorescence titration of the probe Al-II. A good linear relationship between the Al-II fluorescence intensity and Al^3+^ concentration could be obtained in the range from 1 to 5 μM (R = 0.9947). The detection limit was calculated with the equation: Detection limit = 3*y-intercept/slope (as shown in Figure 4), and it was measured to be 0.66 μm.

### 3.4. Job’s Plot

**Figure 5:**
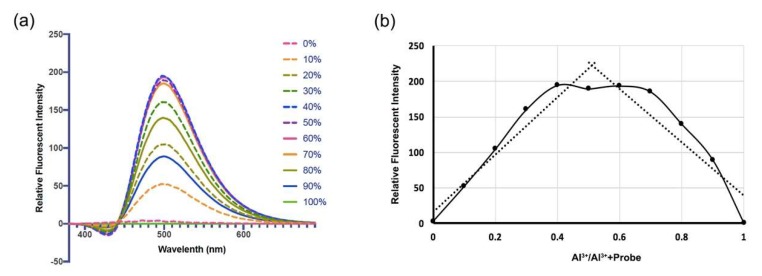
The stoichiometry between the Al-II probe and Al^3+^. (**a**) Relative fluorescent spectra of different combination system of Al^3+^ and Al-II probe (with Al^3+^ from 0% to 100%, the total concentration is 10 μM). (**b**) Job’s plot for determining the stoichiometric complexation of Al-II probe with Al^3+^ in aqueous solution. The fluorescence at 500 nm emission was plotted.

The stoichiometry between the Al-II probe and Al^3+^ was validated by Job’s plot. The fluorescence emission intensity was measured by changing the molar fraction of Al^3+^ (the total concentration was 10 μM) (Figure S7). As shown in Figure 5, the fluorescence emission intensity indicated a maximum at a molar fraction of about 0.5, exhibiting a 1:1 stoichiometry of the probe-Al^3+^ complex.

### 3.5. UV-vis Spectra

**Figure 6:**
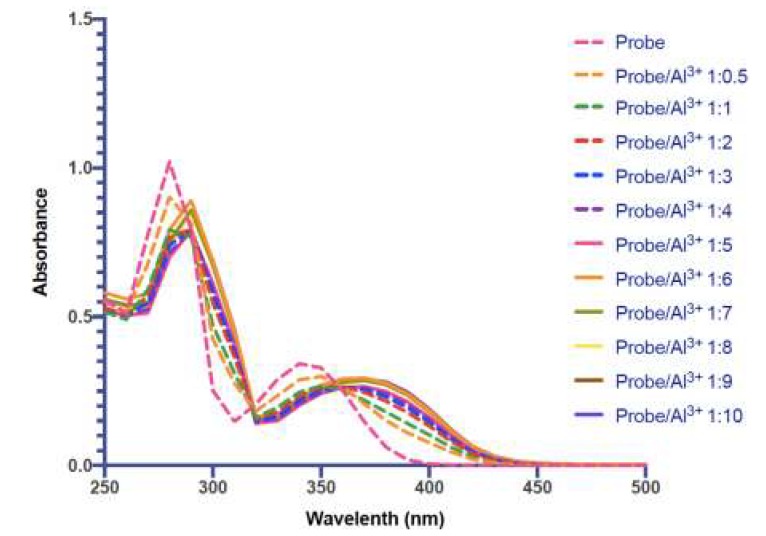
Absorbance spectra of Probe Al-II (25 μM) with titration of increased concentration of Al^3+^ (1.5 μM to 250 μM).

To elucidate the binding mode of the probe and Al^3+^, we have performed the UV-vis spectra test. As shown in Figure 6, probe Al-II (25 μM) has two absorbance peaks at 280 nm and 340 nm. As the Al^3+^ concentration was increasing, the absorption peaks at 280 nm and 340 nm were gradually red-shifted to 290 nm and 370 nm. The intensity of new absorption peaks at 290 nm and 370 nm were increased upon addition of increased concentration of Al^3+^ and the intensity reached to stable value when the concentration of Al^3+^ added was 150 μM (6 equiv.). The large spectral bathochromic shift indicated the deprotonation, as a consequence of Al^3+^ binding to phenol.

We also measured the absorbance spectra of probe in the presence of Cu^2+^ and Fe^3+^ (Figure S8). Compared with Al^3+^, Cu^2+^ gave a larger red-shifted peak around 410 nm and Fe^3+^ has a very broad spectrum, which is consistent with the presence of ligand-to-metal charge-transfer (LMCT) in case of Cu^2+^ and Fe^3+^.

### 3.6. Application of Probe Al-II for Aluminum Detection in Living Cells

**Figure 7:**
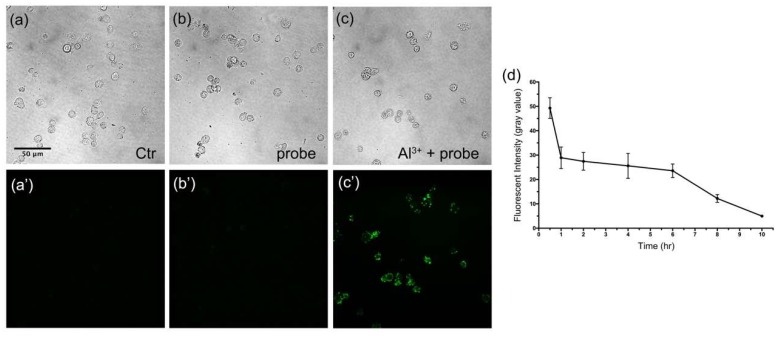
Detection of Al^3+^ in *Drosophila* S2 cells using the Al-II probe upon excitation at 402 nm. (**a**,**a’**) The cells without any treatment. (**b**,**b’**) The cells incubated with the probe (500 μM, 30 min). (**c**,**c’**) The cells sequentially incubated with Al^3+^ (100 mM, 30 min) and the probe (500 μM, 30 min). The fluorescence images (**a’**,**c’**) are corresponding to the bright field images (**a**,**c**), respectively. The horizontal bar in panel a represents 50 μm and applies for all images of this figure. (**d**) The measurement of the fluorescent intensity in Al-probe-treated cells.

We examined the ability of Al-II probe for the detection of intracellular Al^3+^ in living biological systems under a confocal laser scanning microscopy. *Drosophila* S2 cells were incubated in the culture medium containing Al^3+^ (100 mM) for 30 min at room temperature. Then the Al^3+^ medium was removed and cells were washed with PBS buffer solution three times. The cells were further treated with Al-II probe (500 μM) for 30 min at room temperature, followed by PBS washing, three times, and imaging under confocal fluorescence microscope (Figure 7). As a negative control, the cells without Al^3+^ treatment showed no fluorescence (Figure 7a,b). The S2 cells with Al^3+^ treatment exhibited a strong green fluorescence (Figure 7c). Moreover, the cell morphology remained in a good condition after the addition of Al-II probe, indicating low toxicity of the probe. The Al-II probe can also be applied to the fixed cells, giving the similar results. These data demonstrated that the Al-II probe is cell membrane permeable and is useful for the imaging of intracellular Al^3+^ ions in living cells and fixed cells as well.

In addition, we measured the retention time of the Al-probe fluorescence inside cells (Figure 7d). As shown, the fluorescence intensity of Al-probe was quickly decreased after 30 min and maintained at a half level from 1 h to 6 h, then gradually decreased until nearly invisible at 10 h time point.

### 3.7. Imaging of Al^3+^ in Drosophila Malpighian Tubules Using Al-II Probe

**Figure 8:**
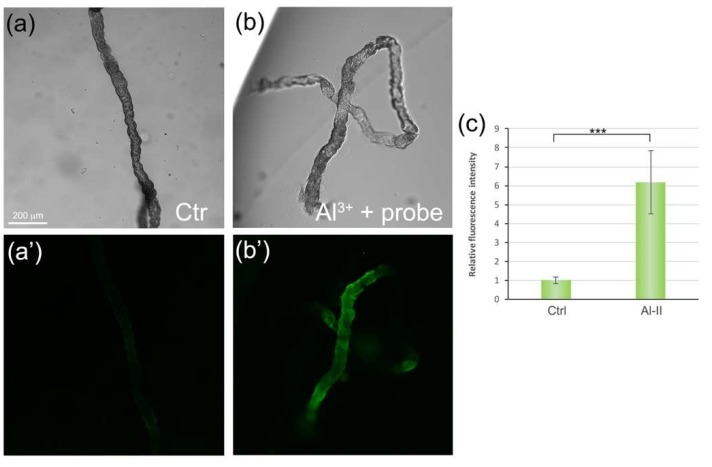
Detection of Al^3+^ in *Drosophila* Malpighian tubules using the Al-II probe upon excitation at 402 nm. (**a**) A bright field image of Malpighian tubules without treatments. (**a’**) The corresponding fluorescence image of Malpighian tubules in panel a. (**b**) A bright field image of Malpighian tubules treated with Al^3+^ (100 mM, 30 min) and probe (1 mM, 30 min). (**b’**) The corresponding fluorescence image of Malpighian tubules in panel b. The horizontal bar in panel a represents 200 μm and applies for all four images of this figure. (**c**) The quantification of relative fluorescence intensity of Al-II probe in Malpighian tubules. *n* >= 3. * represents *p* < 0.05, ** represents *p* < 0.005, *** represents *p* < 0.0005.

The most attractive application for Al-II probe is sensing Al^3+^ in living organisms [31]. We chose the *Drosophila* Malpighian tubules as the model to detect Al^3+^ ions using the Al-II probe in living biological systems. The Malpighian tubule is the main organ to process metal ions in *Drosophila*, functionally analogous to mammalian kidney, and has recently become an important model organism for the study of metal ion transportation [32]. We first fed the 3rd instar larvae with Al^3+^ (100 mM) for 30 min and dissected the Malpighian tubules from larval bodies and washed them with PBS buffer. Then, we treated the Malpighian tubules with the Al-II probe (1 mM) for 30 min at room temperature, followed by image capture using confocal fluorescence microscope (Figure 8). As shown, compared with control sample without treatments, the Malpighian tubules with Al^3+^ and probe treatments showed strong fluorescence. These evidence indicated that the Al-II probe can be used for monitoring the distribution of Al^3+^ in living bodies.

## 4. Conclusions

In summary, we have designed and successfully synthesized a fluorescent probe Al-II to detect Al^3+^ ions. The probe revealed high selectivity and strong fluorescence to Al^3+^. Moreover, we successfully detected the intracellular Al^3+^ in the living cells as well as *Drosophila* Malpighian tubules utilizing the Al-II probe. Therefore, the Al-II probe not only is able to detect Al^3+^ ions in aqueous solution, but also serves as a potentially useful tool for monitoring the intracellular distribution of Al^3+^ in biological systems.

## Figures and Tables

**Table 1:** Summary of the fluorescent probes for aluminum in biological systems.

Mechanism	Application	Reference
FRET	Cultured Cell	[20]
PET	Cultured Cell	[21,22,23]
CHEF	Cultured Cell	[23,24]
AIEE	Cultured Cell	[25]
ESIPT	Cultured Cell	[23,26,27,28]
ESIPT	Bacteria (*E. coli*)	[12]
ESIPT	Plant (rice seedlings)	[29]
ESIPT	Cultured Cell and zebrafish	[13,30]
ESIPT	Cultured Cell and *Drosophila*	Present work

## Supplementary Materials

The following are available online at https://www.mdpi.com/1424-8220/19/11/2423/s1: Figure S1: HPLC spectrum of compound Al-II. Figure S2: ^1^H NMR spectrum of compound Al-II. Figure S3: ^13^C NMR spectrum of compound Al-II. Figure S4: Fluorescent spectra of Al-II probe (10 μM) with titration of increased concentration of Al^3+^ (1 μM to 50 μM) after spectra subtraction of Al-II probe. Figure S5: Nonlinear one-site specific binding curve with relative fluorescent intensity after Al^3+^ (1 μM to 50 μM) titration (R^2^ = 0.9816, Kd = 10.8). Figure S6: Fluorescence response of the probe to Al^3+^ in the presence of higher concentration of Na^+^ and K^+^. Figure S7: Original fluorescent spectra of different combination system of Al^3+^ and probe (with Al^3+^ from 0% to 100%, the total concentration is 10 μM). Figure S8: Absorbance spectra of Probe Al-II (25 μM) with Al^3+^, Cu^2+^ or Fe^3+^ (250 μM).
