# Supplementary material for: A Highly Selective Turn-on Fluorescent Probe for the Detection of Aluminum and Its Application to Bio-Imaging
Source: Sensors (Basel). 2019 May 28;19(11):2423. doi: 10.3390/s19112423 (PMC6603591; doi:10.3390/s19112423)
Supplement: Supplementary file 1 [file sensors-19-02423-s001.pdf]

Supplementary Material

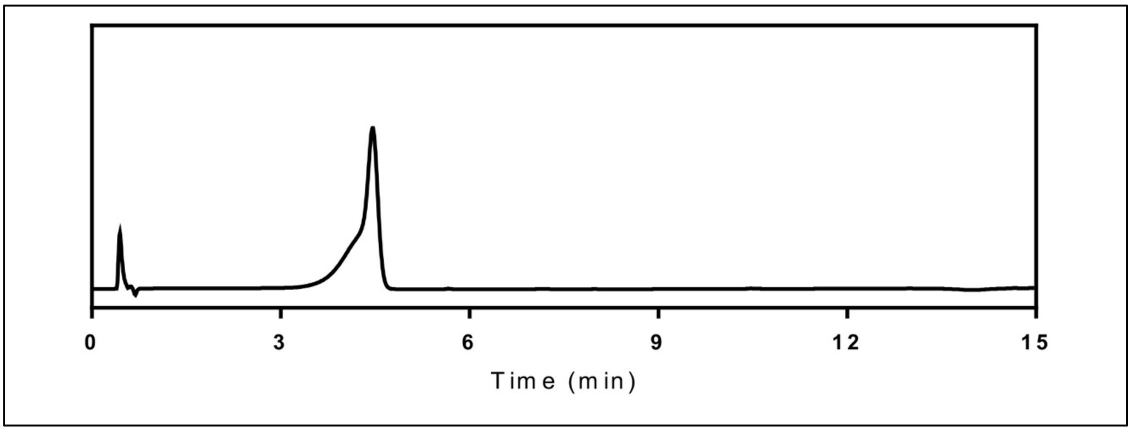

Figure S1. HPLC spectrum of compound **Al-II**

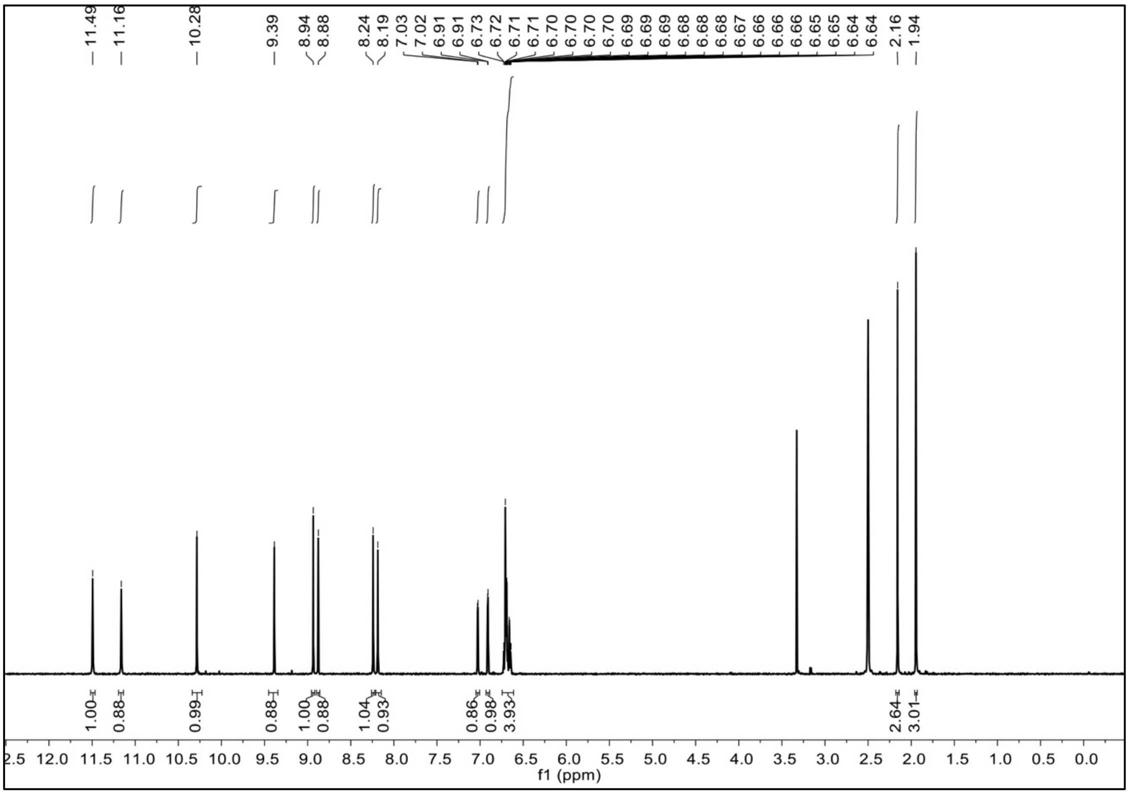

Figure S2. ^{1}H NMR spectrum of compound **Al-II**

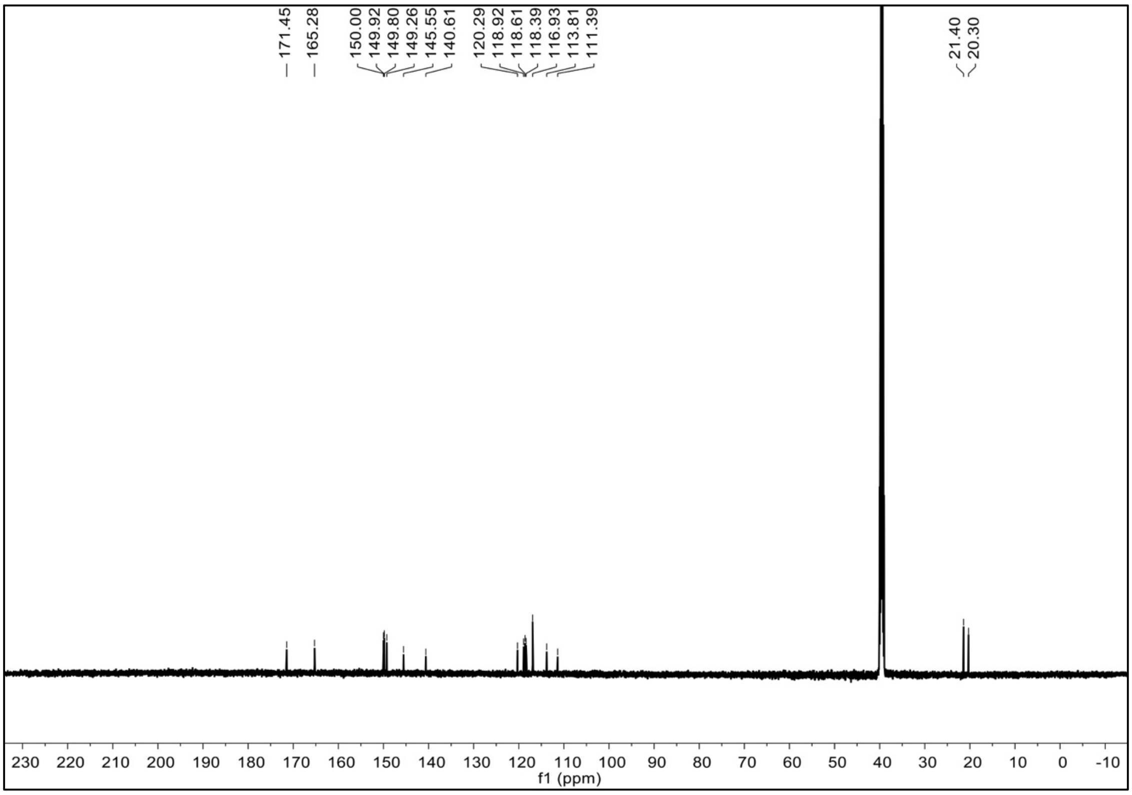

Figure S3. ^{13}C NMR spectrum of compound **Al-II**.

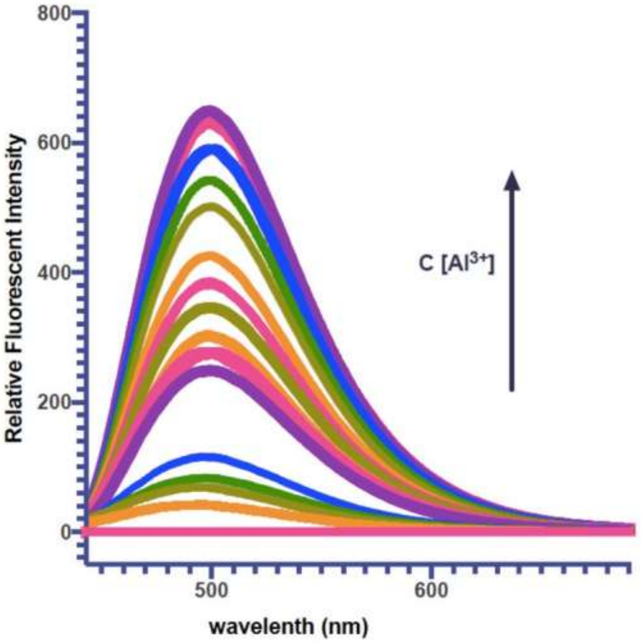

Figure S4. Fluorescent spectra of **Al-II** probe (10 μM) with titration of increased concentration of Al^{3+} (1 μM to 50 μM) after spectra subtraction of **Al-II** probe.

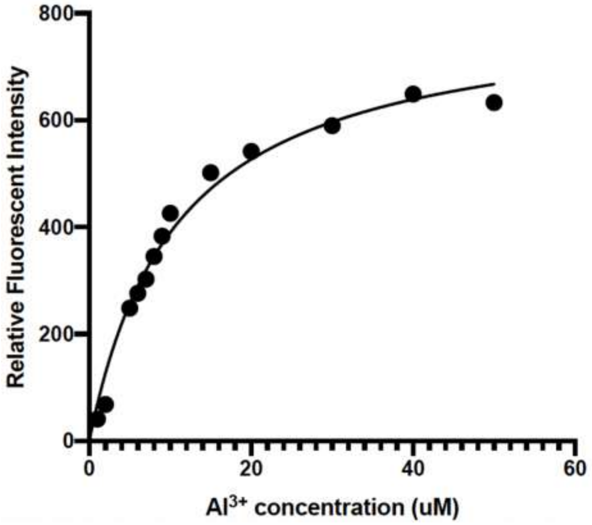

Figure S5. Nonlinear one-site specific binding curve with relative fluorescent intensity after Al^{3+} (1 μM to 50 μM) titration ($R^2 = 0.9816$, Kd = 10.8).

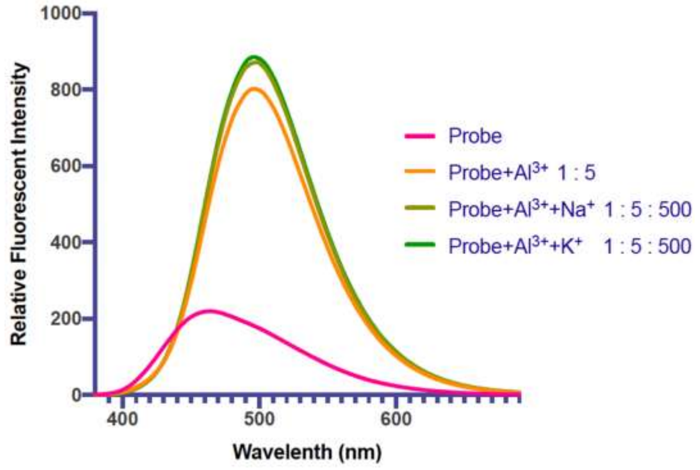

Figure S6. Fluorescence response of the probe to Al^{3+} in the presence of higher concentration of Na^{+} and K^{+}.

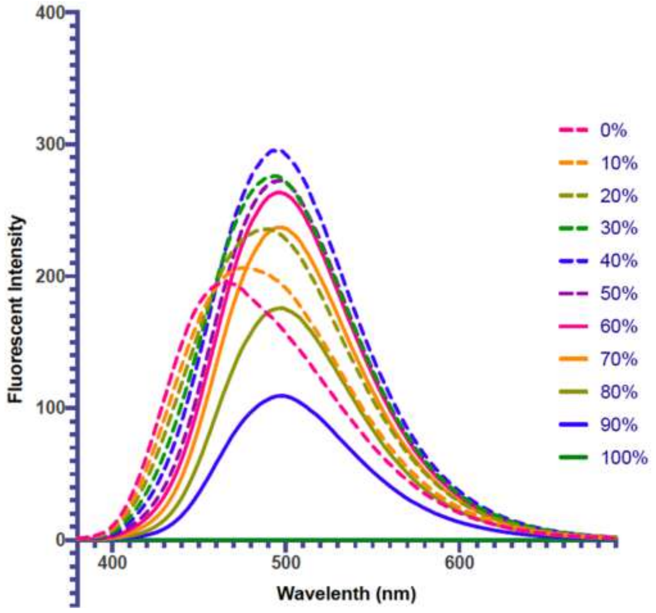

Figure S7. Original fluorescent spectra of different combination system of Al^{3+} and probe (with Al^{3+} from 0% to 100%, the total concentration is 10 μM).

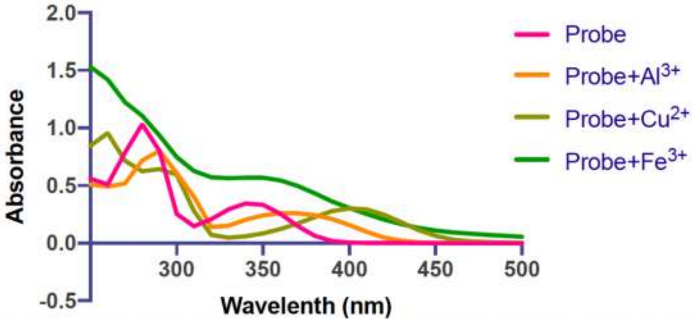

Figure S8. Absorbance spectra of Probe Al-II (25 μM) with Al^{3+}, Cu^{2+} or Fe^{3+} (250 μM).
